# Biological and socioeconomic factors as moderator in relationship between leisure-time physical activity and cardiometabolic risk in adolescents from southern Brazil

**DOI:** 10.1186/s12199-021-01012-5

**Published:** 2021-09-14

**Authors:** Ana Paula Sehn, Debora Tornquist, Luciana Tornquist, Javier Brazo-Sayavera, Cézane Priscila Reuter

**Affiliations:** 1 Graduate Program in Health Promotion, University of Santa Cruz do Sul (UNISC), Av. Independência, 2293, Universitário, Santa Cruz do Sul, Rio Grande do Sul 96815-900 Brazil; 2 Graduate Program in Physical Education, Federal University of Pelotas (UFPel), Pelotas, Rio Grande do Sul Brazil; 3 Department of Sports and Computer Sciences, Universidad Pablo de Olavide, Seville, Spain; 4 Graduate Program in Health Promotion, Department of Health Sciences, University of Santa Cruz do Sul (UNISC), Santa Cruz do Sul, Rio Grande do Sul Brazil

**Keywords:** Physical activity, Metabolic diseases, Sex, Skin pigmentation, Schools, Youth

## Abstract

**Background:**

Given the important repercussions that sociodemographic factors can have on physical activity, especially in the field of leisure, and cardiometabolic risk, it seems relevant to analyze the implications of these variables on the relationship between physical activity in leisure time (LTPA) and cardiometabolic risk. In this sense, the present study aims to verify the moderating role of biologic and socioeconomic factors in the relationship between LTPA and cardiometabolic risk in adolescents in southern Brazil.

**Methods:**

Cross-sectional study that included 1596 adolescents selected at random (58.2% girls), aged between 10 and 17 years. LTPA, biological and socioeconomic factors were assessed using a self-reported questionnaire and the cardiometabolic risk score (total cholesterol/HDL-c ratio, triglycerides, fasting glucose, systolic blood pressure, and waist circumference, considering the participant’s age and sex) was included as an outcome. Associations and moderations were tested by multiple linear regression models.

**Results:**

It was observed a positive interaction of LTPA and sex (*p* = 0.048) and LTPA and school system (*p* = 0.037), and negative interaction of LTPA and skin color (*p* = 0.040), indicating that these factors were moderators in the relationship between LTPA and clustered cardiometabolic risk score (cMetS) in adolescents. A reduction in cardiometabolic risk was observed according to the increase in weekly minutes of LTPA among boys, non-white adolescents, and students from municipal schools.

**Conclusions:**

The association between LTPA and cardiometabolic risk was moderated by sex, skin color, and school system in adolescents from southern Brazil.

## Background

Despite the efforts to increase physical activity levels (PA) in the population and recognition of its important role in maintaining good health and preventing various chronic diseases, the prevalence of insufficient physical activity is a cause for concern worldwide [1]. Globally, most adolescents do not meet current PA guidelines and the prevalence of young people who meet the recommendations has been decreasing over the years [2–4]. In Brazil, 83.6% of adolescents were insufficiently active in 2016 [2].

As well as insufficient physical activity, the prevalence of cardiometabolic risk factors is a concern, and studies have shown the association between them. It was shown that insufficient physically active adolescents have a worse cardiometabolic risk profile when compared to active individuals [5–7] and that insufficient LTPA is an important marker of unfavorable cardiometabolic profile [8, 9]. In addition, the different domains of PA have different associations with health, with LTPA having a more pronounced association with health indicators [10, 11].

It has been shown that sociodemographic factors can influence cardiometabolic risk and LTPA, in which PA levels can differ according to the categories of sex, age, skin color, socioeconomic level (SES), and education system [2, 12, 13]. Thus, given the important repercussions that these factors can have on PA, especially in the field of leisure, and cardiometabolic risk, it seems relevant to analyze the implications of these variables on the relationship between LTPA and cardiometabolic risk. In this sense, the present study aims to verify the moderating role of sociodemographic factors in the relationship between LTPA and cardiometabolic risk in adolescents from southern Brazil.

## Methods

Cross-sectional design study including adolescents aged 10-17 years old, who were students from public and private schools, from urban and rural areas in the municipality of Santa Cruz do Sul, RS (Brazil). The Research Ethics Committee of the University of Santa Cruz do Sul approved the study (Registry Number 1,498,305; Resolution 466/2012). The parents or guardians signed informed consent to allow adolescents to take part in the study.

The sampling process was based on a population of 17,688 students from 50 schools in Santa Cruz do Sul, Rio Grande do Sul (Brazil). Subsequently, a calculation was carried out including the population density of students from all school system (public and private networks), zone (center, north, south, east, and west), and school area (urban and rural) to obtain the total number of individuals to be included in the study. Thus, the 25 participating schools were defined by raffle according to conglomerate extracting, in which all students from 6 to 17 years of age from the selected classes in these schools were invited. Furthermore, it is important to highlight that the population of Santa Cruz do Sul is one of the main centers of German colonization [14], being 86,12% of white skin color [15].

The following inclusion criteria were considered to take part in the current study: (a) be 10 to 17 years old; (b) be enrolled in the school and one of the drawn classes. The exclusion criteria adopted were (a) present intellectual/cognitive deficiencies or limitations to understand and fill out the research instrument or physical limitations that interfere with the evaluations; (b) present data inconsistency or absence of any information regarding the variables included in the study; and (c) not having performed the blood collection.

The sample size calculation was performed in the G * Power 3.1 program (Heinrich Heine Universidad—Düsseldorf, Germany), for the Poisson regression analysis, in which the following reference parameters were considered: test power (1 − β) = 0.95, effect size = 0.15, and significance level α = 0.05, which estimated a minimum sample of 1587 adolescents [16].

Previously trained professionals collected the data at the university during 2016 and 2017. The assessments schedule was set up in advance directly with the schools, and transportation was provided for students’ displacement to the location of assessments. In total, 1917 adolescents participated in the data collection. After applying the exclusion criteria, 321 students were excluded from the sample. Thus, 1596 schoolchildren made up the final sample of the study.

### Collection instruments

Sex, age, skin color, and maturation stage were considered as biological factors and school system, school area, and SES as socioeconomic factors. The data regarding sex, age, skin color, school system, and school area were obtained through a self-reported questionnaire. The evaluation of skin color was considered white, black, brown/mulatto, indigenous, or yellow, posteriorly reclassified in white and non-white (in which all other responses were grouped). The maturation stage was evaluated according to Tanner’s criteria. The evaluation was performed in a specific room, in which only the adolescent and the evaluator remained. The evaluator explained the figures and steps for the adolescents, which included breast development (girls), genital development (boys), and pubic hair (both), and these should indicate their current stage, being classified into prepubertal (stage 1), initial development (stage 2), continuous maturation (stages 3 and 4), and matured (stage 5). Subsequently, the stages were grouped into three categories: prepubertal/initial development, continuous maturation (stages 3 and 4), and matured [17].

The evaluation of the SES followed the criteria of the Brazilian Association of Research Companies (ABEP), considering in the scoring system the items of residence, level of education of the head of the family, and access to public services, being classified according to the sum of points in six SES: A, B1, B2, C1, C2, D-E [18]. For the present study, SES was joined and recategorized into three: upper status (A-B1-B2), medium status (C1-C2), and lower status (D-E).

LTPA was assessed utilizing an adapted self-reported questionnaire by Barros and Nahas [19], based on the question “Do you currently do any sport/ physical activity?”, containing the alternatives “no” and “yes.” The subjects who answered “yes” must indicate the activities performed, the number of weekly sessions, and the duration of each session. Subsequently, the minutes in each activity were added to obtain the total minutes of the week. PA performed during commuting and physical education classes were not considered at the LTPA.

The cardiometabolic risk was assessed using a clustered cardiometabolic risk score (cMetS) that includes the sum of the Z score for each risk factor: total cholesterol (TC)/high-density lipoprotein cholesterol (HDL-c) ratio, triglycerides, fasting glucose, systolic blood pressure (SBP), and waist circumference, considering the participant’s age and sex. Cardiometabolic risk score calculation was performed using the following formula: *Z* score ([value of a continuous variable – cut-off points]/standard deviation), using the cut-off points and standard deviation determined by Stavnsbo et al. [20]. The *Z* score of HDL-c that has an inverse relationship with the cardiometabolic risk was multiplied by −1 [21].

TC, HDL-c, triglycerides, and glucose were assessed from vein blood collected after 12-h fasting. Serum samples were analyzed using commercial Kovalent/DiaSys kits (DiaSys Diagnostic Systems, Germany) in an automatic analyzer (Miura 200, I.S.E., Rome, Italy). Blood pressure was measured using the auscultatory method on the left arm with a previous rest of 5 min before the measurement. Two measures were recorded, registering the one with the lowest SBP. To assess waist circumference, inelastic tape with 1 mm resolution was used and the narrowest part of the trunk was considered, between the last rib and the iliac crest [22].

### Statistical analysis

Statistical analysis was performed using the Statistical Package for Social Sciences (SPSS) software version 23.0 (IBM Corp). Data were described through the distribution of the relative and absolute frequencies of the studied exposures and outcomes, as well as means and standard deviations (SD) and median and interquartile interval (II). For the moderation analyses, the PROCESS macro for the SPSS through multiple linear regression models was used. It was tested with the following relationships in each model: the direct association of the independent variable (LTPA) on the dependent variable (cMetS); the moderator variable (biological and sociodemographic factors) on cMetS; and the interaction association between the independent variable and the moderator variable. The Johnson–Neymann technique was applied to determine the associations between the independent and dependent variables in all categories of the moderator’s variables. The LTPA points presented in the figure were determined by 16th, 50th, and 84th percentiles. A hierarchical conceptual model was used to include variables in the adjusted analyses. In the first level, biological variables (sex, skin color, age, and sexual maturity) were included, and in a second level, socioeconomic variables (family economic class, education network, and school area), with the model adjusted for the variables of the same level and the previous level of the moderating variable. An alpha ≤ 0.05 was adopted.

## Results

A total of 1596 adolescents composed the sample, 58.20% were female, 60.20% were in the 13 to 17 age group and 77.90% were white skin color. Table 1 describes sample characteristics. The median LTPA was 90 min per week (II, 0 to 240 min).

**Table 1:** Descriptive characteristics in adolescents aged 10 to 17 years in the municipality of Santa Cruz do Sul, 2016 and 2017 (*n* = 1596)

	***n*** (%)
**Sex**	
Male	667 (41.80)
Female	929 (58.20)
**Age groups**	
10 to 12 years	636 (39.80)
13 to 17 years	960 (60.20)
**Skin color**	
White	1243 (77.90)
Nonwhite	353 (22.10)
**Maturational stage**	
Prepubertal/initial	466 (29.20)
Continuous maturation	943 (59.10)
Matured	187 (11.70)
**Socioeconomic status**	
Upper	620 (38.80)
Middle	844 (52.90)
Lower	132 (8.30)
**School system**	
Municipal	406 (25.40)
State	1059 (66.40)
Private	131 (8.20)
**School area**	
Urban	1373 (86.00)
Rural	223 (14.00)
	**Mean (SD)**
Age (years)	13.25 (2.02)
TC (mg/dL)	159.98 (31.65)
HDL-c (mg/dL)	57.08 (10.33)
TC/HDL-c ratio	2.86 (0.64)
Triglycerides (mg/dL)	71.58 (32.59)
Fasting glucose (mg/dL)	88.54 (6.95)
Systolic blood pressure (mmHg)	107.81 (13.227)
Waist circumference (cm)	67.78 (9.58)
cMetS (score)	−0.10 (0.63)
	**Median [II]**
LTPA (min/week)	90 [0-240]

*SD* standard deviation, *n* absolute frequency, *%* relative frequency, *II* interquartile interval, *TC* total cholesterol, *HDL-c* high-density lipoprotein cholesterol, *TC/HDL-c* cholesterol/high-density lipoprotein cholesterol ratio, *cMetS* clustered cardiometabolic risk score, *LTPA* leisure-time physical activity

The biological factors, sex, skin color, sexual maturation, and age groups, were tested as moderators in the relationship between LTPA and cMetS and are shown in Table 2. It was observed a positive interaction of LTPA and sex and negative interaction of LTPA and skin color, indicated that sex and skin color were moderators in the relationship between LTPA and cMetS (*p* = 0.044; *p* = 0.040, respectively) in adolescents.

**Table 2:** Biological factors as a moderator in the relationship between LTPA and cMetS in adolescents

	cMetS	
	***β*** (95% CI)	***p***
**Model 1**		
**LTPA**	−0.00067 (−0.00112; −0.00022)	0.003
**Sex**		
Male	REF	
Female	0.02819 (−0.05149; 0.10787)	0.495
**LTPA × sex**	0.0029 (0.00001; 0.00058)	0.044
**Model 2**		
**LTPA**	0.00019 (−0.00025; 0.00063)	0.393
**Skin color**		
White	REF	
Nonwhite	−0.04011 (−0.13201; 0.05179)	0.392
**LTPA × skin color**	−0.00036 (−0.00070; −0.00002)	0.040
**Model 3**		
**LTPA**	−0.00032 (−0.00060; −0.00004)	0.024
**LTPA/maturational stage**		
Prepubertal/initial	REF	
Continuous maturation	0.11384 (0.02205; 0.20564)	0.015
Matured	0.19105 (0.04662; 0.33548)	0.010
**LTPA × maturational stage**		
LTPA **×** prepubertal/initial	REF	
LTPA **×** continuous maturation	0.00012 (−0.00021; 0.00046)	0.463
LTPA **×** matured	0.00002 (−0.00046; 0.00050)	0.936
**Model 4**		
**LTPA**	−0.00011 (−0.00062; 0.00041)	0.684
**Age groups**		
10 to 12 years	REF	
13 to 17 years	0.00392 (−0.08093; 0.08877)	0.928
**LTPA × age groups**	−0.00008 (−0.00038; 0.00022)	0.584

*β* linear regression coefficient, *CI* confidence interval, *LTPA* leisure-time physical activity, *cMetS* clustered cardiometabolic risk score, *REF* reference category. Model 1: adjusted for skin color, maturational stage, and age. Model 2: adjusted for sex, maturational stage, and age. Model 3: adjusted for sex, skin color, and age. Model 4: adjusted for sex, skin color, and maturational stage

The moderator role of socioeconomic factors in the relationship between LTPA and cMetS in adolescents is presented in Table 3. Positive interaction between LTPA and school system state (*p* = 0.037), in comparison with school system municipal, indicated that school system act as a moderator in the relationship between LTPA and cMetS.

**Table 3:** Socioeconomic factors as a moderator in the relationship between LTPA and cMetS in adolescents

	cMetS	
	***β*** (95% CI)	***p***
**Model 1**		
**LTPA**	−0.00032 (−0.00053; −0.00010)	0.004
**Socioeconomic status**		
Upper	REF	
Middle	−0.00056 (−0.08548; 0.08435)	0.990
Lower	−0.08158 (−0.23571; 0.07256)	0.299
**LTPA × socioeconomic status**		
LTPA **×** upper	REF	
LTPA **×** middle	0.00011 (−0.00018; 0.00040)	0.462
LTPA **×** lower	0.00031 (−0.00030; 0.00092)	0.323
**Model 2**		
**LTPA**	−0.00052 (−0.00082; −0.00022)	0.001
**School system**		
Municipal	REF	
State	−0.14094 (−0.23415; −0.04773)	0.003
Private	−0.12554 (−0.30296; 0.05189)	0.165
**LTPA × school system**		
LTPA **×** municipal	REF	
LTPA **×** state	0.00037 (0.00002; 0.00072)	0.037
LTPA **×** private	0.00019 (−0.00038; 0.00076)	0.519
**Model 3**		
**LTPA**	−0.00024 (−0.00076; 0.00028)	0.362
**School area**		
Urban	REF	
Rural	−0.01034 (−0.12454; 0.10386)	0.859
**LTPA × school area**	0.00001 (−0.00045; 0.00045)	0.993

*β* linear regression coefficient, *CI* confidence interval, *LTPA* leisure-time physical activity, *cMetS* clustered cardiometabolic risk score, *REF* reference category. Model 1: adjusted for a school system, school area, sex, skin color, maturational stage, and age. Model 2: adjusted for socioeconomic status, school area, sex, skin color, maturational stage, and age. Model 3: adjusted for socioeconomic status, school system, sex, skin color, maturational stage, and age

**Fig. 1:**
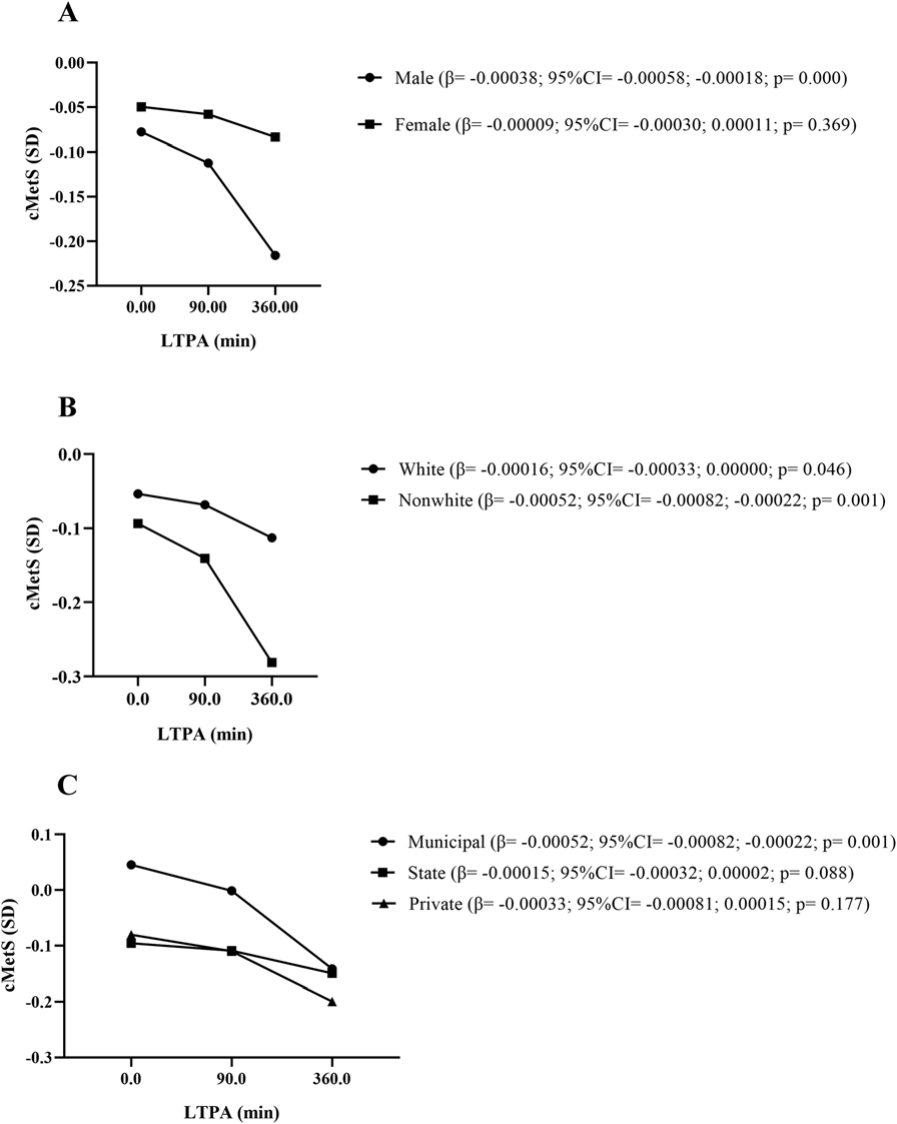
Moderation of sex, skin color, and school system in the relationship between LTPA and cMetS. **A** moderator role of sex; **B** moderator of skin color; **C** moderator role of school system; LTPA, leisure-time physical activity; cMetS, clustered cardiometabolic risk score; β, linear regression coefficient; CI, confidence interval; SD, standard deviation

The Johnson–Neymann technique was applied for the variables that demonstrated a significant interaction with LTPA, in which the association between LTPA and cMetS was modified according to categories of sex, skin color, and school system (Fig. 1). Regarding sex, only among boys is observed an association between LTPA and cMetS, in which there is a linear reduction in cardiometabolic risk as the weekly minutes of LTPA increase (Fig. 1A). Moderation by skin color was also observed, with a linear association between LTPA and cardiometabolic risk occurring in non-white adolescents, in which there is a reduction in cMetS according to the increase in the weekly time of LTPA (Fig. 1B). Finally, there is also moderation on the part of the education system, with the relationship between LTPA and cMetS occurring only among students from municipal schools, in which, as the time spent on LTPA for adolescents increases, there is a reduction in cardiometabolic risk (Fig. 1C).

It is also possible to observe that among boys, non-white adolescents, and students from municipal schools, from about 90 min a week of LTPA, there is a greater strength of association, with a more accentuated drop in cardiometabolic risk, being possible to observe a dose-response in which, as the weekly minutes spent on LTPA increase, the lower the levels of cMetS in these groups (Fig. 1).

## Discussion

The present study indicated that sex, skin color, and school system moderated the relationship between LTPA and cMetS in schoolchildren from southern Brazil. Among male adolescents, non-white skin color, and students from the municipal school system, there is a linear reduction in cardiometabolic risk according to the increase in weekly minutes of PA, with a more accentuated reduction from about 90 min per week of LTPA, with an observable dose-response.

Regarding gender, the result that only the male sex was shown to influence the relationship between LTPA and cMetS may be explained by two main aspects. First, the biological differences of sex in body composition can influence health risk factors, in which while girls develop more total body fat and have a small gain in lean mass at this stage of life, boys have greater development of lean muscle mass and total, with little increase in body fat [23]. This difference can lead to cardiometabolic risk becoming more expressive among girls, in line with what was shown in the evaluated sample, in which, regardless of the time of LTPA, girls show a higher average of cMetS. Second, the girls’ behavioral aspects are also more favorable to the development of risk factors, considering that it has been shown that Brazilian girls have a higher occurrence of health risk behavior [24–26], including among these are the fact that they are more inactive than boys [2, 27–29]. Furthermore, the sexes can show divergent physiological responses to physical activity, with boys showing more positive responses to health concerning moderate and vigorous-intensity activities [30], in addition to the fact that boys present higher levels of PA at these intensities [31–34]. These sexual differences in PA practice can also be due to biological factors, in which the growth spurt and pubertal maturation can influence the interests and habits of boys and girls [35] but sociocultural factors seem to exert the greatest influence. The social roles assigned to gender influence the choice of girls and boys; and girls are raised with less freedom, with greater family restrictions and concerns about safety [36, 37], factors that interfere in the choices and opportunities for leisure practices. All of these factors can influence the relationship of LTPA with cardiometabolic risk and help to understand the gender differences in this association.

Moderation in skin color was also observed, with a linear reduction between LTPA and risk occurring in non-white adolescents. Biological and economic aspects can explain this moderation. White youth tend to have a higher percentage of body fat, especially visceral fat, than black youth, and visceral fat presents a higher risk for metabolic diseases [23]. In addition, a previous study with schoolchildren in the city showed that those who declared themselves black, mulatto, or brown had longer telomere length, a factor that has been pointed out to help explain the ethnic differences in disease risks [38]. Shorter telomeres have been associated with increased body mass index, adiposity, visceral fat accumulation, and other metabolic factors [39]. In addition, active individuals, regardless of the intensity of physical activity, have greater telomere length compared to inactive individuals, indicating the importance of physical activity in reducing oxidative stress and inflammation that affect telomeres [40]. Therefore, this may be a plausible biological explanation to explain higher cMetS levels among white adolescents in the evaluated sample, regardless of the weekly duration of the LTPA and the influence of skin color on the relationship between LTPA and cardiometabolic risk.

Regarding the economic factor, in Brazil, skin color is an important marker of economic inequality, especially disfavoring blacks [41]. It is known that less favorable economic conditions can hinder access to the practice of LTPA. Furthermore, skin color can be related not only to economic conditions but also to other aspects that may contribute to LTPA disparities, such as aspects related to segregation [42]. Also, data from the National Health Survey of Brazil showed that less healthy habits are associated with non-white skin color [43, 44]. Thus, due to the possible set of less healthy lifestyle habits identified among the non-white population, adolescents with this characteristic who practice some LTPA may have a greater potential for change in cardiometabolic risk in response to PA, which may explain the relationship of LTPA with reduced cardiometabolic risk only in this group.

Our results also demonstrated a moderating role of the school system, suggesting that among municipal school students, a linear relationship is observed between the weekly minutes of LTPA and the reduction in cardiometabolic risk. In this context, it is important to note that students from municipal schools had higher cMetS, regardless of time spent in LTPA. We hypothesize that the moderating role found for the municipal school system may be associated with cultural and behavioral aspects of the environment in which these adolescents are inserted. In addition, the municipal education network can be considered an economic marker of students, since in Brazil attending public school is associated with a lower family income [24]. Thus, perhaps the practice opportunities are associated with the school context, with fewer opportunities for PA and extracurricular sports. The relationship between participation in extracurricular activities and higher levels of PA is demonstrated in the literature [45], and studies have pointed out the influence of the school environment on adolescents’ physical activity [46]. However, this hypothesis still needs to be confirmed by further studies.

In addition to PA, other factors such as diet, less healthy lifestyle habits, and health information may be associated with the economic relationship of the school network. In addition, another study realized the same population observed that children and adolescents of the municipal school presented more prevalence of worse cardiorespiratory fitness levels compared with students of private schools [47]. It is also important to point out among the results observed that precisely in the educational network that showed higher average metabolic risk scores, the increase in time spent on LTPA was related to the reduction in risk. This factor is relevant and demonstrates the importance of employing interventions aimed at making students more active, especially in this more vulnerable group and that presents a more evident metabolic risk.

Although it was not observed, a moderating role of economic status, skin color, and educational system are variables that are strongly influenced by economic factors and that played a moderating role in the present study. We hypothesize that the lack of association for the economic status may be due to the economic index being a more subjective and specific measure, in which the quantity of material goods is considered, while skin color and the educational network are broader factors that encompass cultural, environmental, and behavioral factors and not just economic conditions.

The inverse relationship of PA with cardiometabolic risk indicators in adolescents has already been reported in the literature [8, 9, 48–51]. This relationship observed in the young population alerts to the early occurrence of comorbidities related to physical inactivity and that, in the case of modifiable behavior, could be avoided. Evidence of a decline in PA levels among young people warns of the importance of interventions in the area [28]. In addition, the existing evidence that PA in childhood and adolescence influences the health and practice of PA in adulthood [52] reinforces the importance of encouraging PA practice among the young population.

Furthermore, the study showed that in between boys, non-whites, and students from municipal schools, the greater strength of association is observed after approximately 90 min per week of LTPA, with a more marked reduction in cardiometabolic risk as the weekly minutes of LTPA increase, demonstrating a dose-response relationship. Corroborating our findings, a systematic review indicated a dose-response relationship between PA and health benefits, in which as PA increases, the better the individuals’ health conditions [53]. However, the study demonstrated that even modest amounts of PA can bring positive health outcomes in at-risk youth, such as the obese [53]. Another study also demonstrated an improvement in the cardiometabolic risk profile over time, as the time allocated for PA increased [54].

The World Health Organization recommends that adolescents perform at least 60 min/day of moderate and vigorous PA, which corresponds to a weekly sum of 420 min [55]. In that regard, our results may indicate that doing some PA, even not meeting the recommendations, maybe enough to promote cardiometabolic health. Previous evidence has shown that remaining physically active, even without meeting PA guidelines, is relevant for preventing cardiometabolic risk in this population [6, 7]. In this context, although the evidence of a favorable relationship between PA and cardiometabolic risk is more consistent and robust concerning moderate and vigorous PA, less intense PA also demonstrates benefits [50, 56]. In addition, all PA patterns are beneficial, whether performed in sporadic or continuous sessions, in different contexts, settings, and intensities [50, 56]. Thus, when thinking about PA in a context of health promotion and prevention of cardiometabolic risk, every activity is important, in which PA can be accumulated in small doses throughout the day to obtain health benefits, which meets the highlights of the new WHO guidelines that recommend that “all movement is important” [55].

One of the main limitations of this study is that the evaluation of LTPA through a questionnaire, limiting the assessment to the time of practice and not measuring the intensity. In addition, it is known that this method of evaluation is limited by temporality, memory bias of respondents, social desirability, and classification errors. However, the evaluation of PA by questionnaire is widely accepted in population studies, and, our sample is probably accurate to support our results. As with any cross-sectional study, it is not possible to attribute causality to the association observed between insufficient physical activity and cardiometabolic risk, although it seems more biologically plausible that insufficient physical activity precedes metabolic risk. In addition, our PA measures were limited to LTPA, not considering activities performed in other domains, such as physical education classes and commuting. Even though various confounding factors have been controlled in the analyses, the possibility of residual confusion exists, due to other factors not included.

The analysis of the moderation used allowed highlighting different categories of time spent in LTPA, unlike the categories usually used based on recommendations. As far as we know, our study is the first to use this type of moderation analysis to assess the influence of biological and socioeconomic factors on the relationship between LTPA and cardiometabolic risk in middle-income countries. As a strong point, the present study presents a representative and accurate sample of adolescents from a city in the interior from southern Brazil, differing from most of the studies conducted in capitals and large centers. In addition, it includes students from all education networks. The use of a continuous risk score to assess cardiometabolic risk should be highlighted. There is evidence indicating that a cluster cardiometabolic risk score is more relevant to future health than risk factors assessed individually [20, 57]. It is suggested that future studies will contrast the current findings, carrying out, for example, analyses with prospective data that can help to clarify and infer causality in the observed associations, using objective measures of PA, allowing to measure the intensity. Furthermore, studies with lifestyle interventions can help confirm whether, when practice levels are increased, cardiometabolic risk can be reduced.

## Conclusions

The moderator role of sociodemographic factors in the relationship between LTPA and cMetS indicated that boys, individuals with non-white skin color, and students at municipal schools showed a reduction in cMetS as they increase the weekly minutes of LTPA. The results of this study bring important elements to understand the existing disparities in the relationship between cardiometabolic risk and LTPA. This study highlights the urgency of actions that effectively increase the involvement of adolescents with PA and the need to develop public policies and PA programs that prioritize the most vulnerable population groups, aiming to promote greater equity in the opportunities of access and choice for adolescents, increasing the chances of raising PA levels in the young population and reducing the risk of cardiometabolic diseases.

## Data Availability

The database used and analyzed in the present study is not publicly available as its information may compromise the participants’ privacy and consent involved in the research. However, the data are available from the corresponding author, upon request.

## Abbreviations

CI: Confidence interval
cMetS: Clustered cardiometabolic risk score
HDL-c: High-density lipoprotein cholesterol
II: Interquartile interval
LTPA: Physical activity in leisure time
PA: Physical activity levels
SBP: Systolic blood pressure
SD: Standard deviations
SES: Socioeconomic level
SPSS: Statistical Package for Social Sciences
TC: Total cholesterol
β: Linear regression coefficient
